# PPARβ/δ: A Key Therapeutic Target in Metabolic Disorders

**DOI:** 10.3390/ijms19030913

**Published:** 2018-03-20

**Authors:** Xavier Palomer, Emma Barroso, Javier Pizarro-Delgado, Lucía Peña, Gaia Botteri, Mohammad Zarei, David Aguilar, Marta Montori-Grau, Manuel Vázquez-Carrera

**Affiliations:** 1 Pharmacology Unit, Department of Pharmacology, Toxicology and Therapeutic Chemistry, Institute of Biomedicine of the University of Barcelona (IBUB), Faculty of Pharmacy and Food Sciences, University of Barcelona, 08028 Barcelona, Spain; xpalomer@ub.edu (X.P.); ebarroso@ub.edu (E.B.); jpizarro@ub.edu (J.P.-D.); lucia.pena.moreno@gmail.com (L.P.); gaia.btt@gmail.com (G.B.); biotech.zarei@gmail.com (M.Z.); d.aguilarrecarte@gmail.com (D.A.); mmontori@ub.edu (M.M.-G.); 2 Pediatric Research Institute-Hospital Sant Joan de Déu, 08950 Esplugues de Llobregat, Spain; 3 Spanish Biomedical Research Center in Diabetes and Associated Metabolic Diseases (CIBERDEM), Instituto de Salud Carlos III, C/Monforte de Lemos 3-5, Pabellón 11, Planta 0, 28029 Madrid, Spain

**Keywords:** PPARβ/δ, obesity, dyslipidaemia, type 2 diabetes mellitus, non-alcoholic fatty liver disease

## Abstract

Research in recent years on peroxisome proliferator-activated receptor (PPAR)β/δ indicates that it plays a key role in the maintenance of energy homeostasis, both at the cellular level and within the organism as a whole. PPARβ/δ activation might help prevent the development of metabolic disorders, including obesity, dyslipidaemia, type 2 diabetes mellitus and non-alcoholic fatty liver disease. This review highlights research findings on the PPARβ/δ regulation of energy metabolism and the development of diseases related to altered cellular and body metabolism. It also describes the potential of the pharmacological activation of PPARβ/δ as a treatment for human metabolic disorders.

## 1. Introduction

Acquired metabolic disorders, particularly obesity and its associated co-morbidities currently pose a risk to human health on a global scale. These metabolic disorders are closely related to adipose tissue dysfunction, one of the primary defects observed in obesity that may link this condition to its co-morbidities such as non-alcoholic fatty liver disease (NAFLD), atherogenic dyslipidaemia, type 2 diabetes mellitus and cardiovascular disease [1,2,3]. In fact, up to a third of obese subjects are metabolically healthy and they do not develop obesity-related metabolic or cardiovascular disorders [4], probably because of the preservation of normal adipose tissue architecture and function. Adipose tissue dysfunction in obese patients results from the interactions of genetic and environmental factors that lead to the presence of hypertrophic adipocytes, which have a pro-inflammatory, insulin-resistant phenotype compared to small adipocytes [5]. In addition, a critical factor contributing to the difference between metabolically healthy and unhealthy obese individuals is the anatomical distribution of the adipose tissues. Expansion of the visceral adipose tissue, which is considered a dysfunctional adipose tissue unable to store excessive levels of lipids, to a greater extent than that of subcutaneous adipose tissue, is associated with metabolic alterations [6]. Failure to store surplus lipids into visceral adipose tissue causes a chronic elevation of circulating fatty acids (FA), which can reach toxic levels in non-adipose tissues, such as skeletal muscle, the liver and the pancreas [7]. The deleterious effect of lipid accumulation in non-adipose tissues is known as lipotoxicity. This surplus of fatty acids (FAs), especially saturated FA and their derived metabolites, such as diacylglycerol and ceramides, induces chronic low-grade inflammation and has harmful effects on multiple organs and systems.

Given the enormous stress on global health services caused by the increasing incidence of obesity and its co-morbidities on global health services, there is a need to better understand the mechanisms behind the relationship between obesity and the development of metabolic disorders to prevent and to improve the outcomes of these diseases. Peroxisome proliferator-activated receptor (PPAR)β/δ is a nuclear receptor that exerts many metabolic effects. Its activation may prevent and improve the outcome of obesity-related metabolic disorders. In this review, we will summarize the molecular features of PPARβ/δ and the benefits of using its agonists to treat obesity and its related co-morbidities.

## 2. Basic Overview of the Molecular Features of PPARβ/δ

PPARβ/δ is a member of the nuclear receptor (NR) superfamily of ligand-inducible transcription factors and belongs to the PPAR family, which comprises three isotypes: PPARα (NR1C1, according to the unified nomenclature system for the NR superfamily); PPARβ/δ (NR1C2); and PPARγ (NR1C3) [8,9]. PPARβ/δ was initially called PPARβ when it was first cloned in *Xenopus laevis*. However, when cloned in other species it was not clearly identified as being homologous to the Xenopus PPARβ and it was alternatively called NUC-1 in humans and PPARδ in mice. Currently, it is accepted that Xenopus PPARβ is homologous to murine PPARδ, giving rise to the terminology PPARβ/δ [8]. PPARβ/δ consists of four major functional domains: The N-terminal ligand-independent transactivation domain (A/B domain), often known as activation function 1 (AF-1); the DNA binding domain (DBD or C domain); the hinge region (D domain); and the carboxy-terminal E domain or AF-2, which includes the ligand-binding domain and the ligand-dependent transactivation domain [8,9]. The major physiological functions of PPARβ/δ result from its activity as a transcription factor, modulating the expression of specific target genes. Through this mechanism, PPARβ/δ regulates lipid metabolism and glucose homeostasis [8,9,10,11]. In addition, PPARβ/δ can regulate inflammation [12]. The involvement of PPARβ/δ in all these functions depends on its tissue distribution, ligand binding and the recruitment of co-activators or co-repressors.

PPARβ/δ is ubiquitously expressed, although it is most abundant in metabolically active tissues, especially in those organs/cells associated with FA metabolism, such as skeletal and cardiac muscle, hepatocytes and adipocytes. It has also been particularly characterized in macrophages. Compared to other NRs, PPARs present a large ligand-binding pocket (≈1300 Å^3^) [8], which directly contributes to the ability of PPARs to bind a great variety of endogenous and synthetic ligands. FAs are considered endogenous PPAR ligands but they show little selectivity for the different PPAR isoforms. Although all-trans retinoic acid has been reported to be a PPARβ/δ agonist [13], this has not been confirmed by other groups [14,15] and therefore remains controversial. To elucidate PPARβ/δ functions, synthetic ligands with high affinity and specificity (GW501516, GW0742 and L-165041) that only activate PPARβ/δ at very low concentrations both in vivo and in vitro have been developed [8]. At present, there are no clinically available drugs targeting PPARβ/δ but three PPARβ/δ agonists have reached clinical trials: Seladelpar (MBX-8025) (CymaBay Therapeutics) [16]; KD-3010 (Kalypsys) [17]; and CER-002 (Cerenis).

To activate transcription, PPARβ/δ forms an obligate heterodimer with retinoid X receptor (RXR or NR2B) and binds to peroxisome proliferator response elements (PPREs) located at the promoter regions of target genes, thereby increasing gene transcription in a ligand-dependent manner (transactivation) [8]. In the absence of a ligand, the PPARβ/δ-RXR heterodimer is bound by nuclear co-repressor proteins, which block transcriptional activation by preventing the binding of the heterodimer to the promoter. Ligand binding induces a conformational change within PPARβ/δ, resulting in the dissociation of the co-repressors and the recruitment of co-activators, which subsequently lead to PPARβ/δ-RXR binding to PPREs to initiate transcription [8]. PPARβ/δ also regulates gene expression independently of DNA binding, via cross-talk with other types of transcription factors, thus influencing their function through a mechanism termed receptor-dependent transrepression [8]. Most of the anti-inflammatory effects of PPARs probably occur through this mechanism [12].

## 3. PPARβ/δ as a Major Regulator of Metabolic Disorders

### 3.1. Obesity

**Figure 1:**
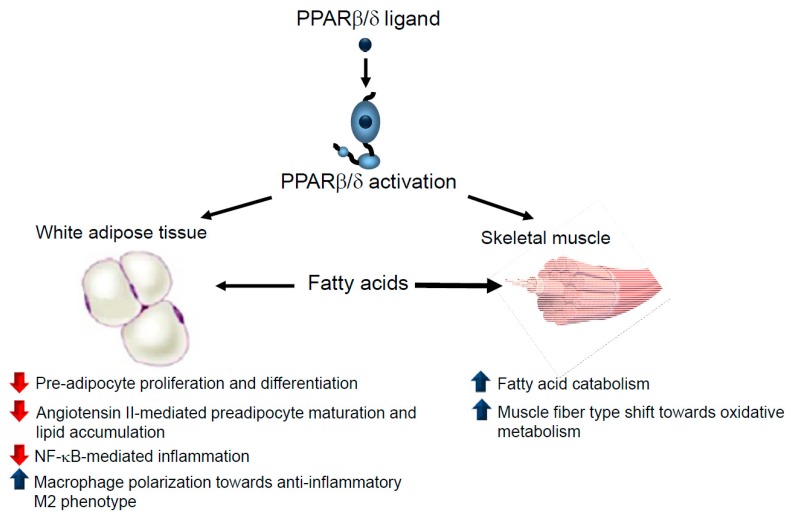
PPARβ/δ activation prevents obesity through several mechanisms. PPARβ/δ activation reduces pre-adipocyte proliferation and differentiation, attenuates angiotensin II-mediated dysfunctional hypertrophic adipogenesis and inhibits inflammation in adipose tissue. PPARβ/δ ligands reduce the availability of fatty acids to be stored in adipose tissue since these drugs induce fat burn in skeletal muscle by either increasing fatty acid oxidation or switching muscle fibre type towards oxidative metabolism. Blue arrow: increases. Red arrow: decreases.

PPARβ/δ-deficient mice exhibited a marked reduction in adiposity compared to wild-type mice levels [18]. This effect, however, cannot be reproduced in mice harbouring an adipose tissue-specific deletion of PPARβ/δ, indicating that PPARβ/δ elicits peripheral functions in systemic lipid metabolism. In fact, PPARβ/δ activation prevents weight gain in diet- or genetically-induced animal models of obesity by increasing fat burning in different tissues [19,20] or switching muscle fibre type, which, in turn, increases the muscle oxidative capacity [21] (Figure 1). A recent study also suggests that intestinal PPARβ/δ protects against diet-induced obesity, since intestinal epithelial cell-specific deletion of PPARβ/δ in mice results in increased amounts of omental white adipose tissue [22].

Additional PPARβ/δ-mediated mechanisms can also contribute to the reduction in adiposity, since PPARβ/δ also regulates preadipocyte proliferation and differentiation through different mechanisms [23,24,25], such as by regulating of the expression of PPARγ, a key regulator of terminal adipocyte differentiation. Moreover, PPARβ/δ ligands prevent angiotensin II-induced adipocyte growth and lipid accumulation [26]. Angiotensin II increases levels of reactive oxygen species (ROS), which attenuate the canonical Wnt signalling pathway, leading to dysfunctional hypertrophic adipogenesis. PPARβ/δ agonists prevent oxidative stress and the reduction in Wnt signalling pathway induced by angiotensin II by increasing the expression of heme oxygenase 1 in adipose tissue. Consequently, PPARβ/δ activation delays preadipocyte maturation and lipid accumulation, leading to increased numbers of smaller adipocytes with an improved adipocytokine profile. Thus, overall, PPARβ/δ activation prevents oxidative stress and dysfunctional adipogenesis under conditions of overactive renin-angiotensin system [26].

In humans, PPARβ/δ expression is reduced in both the subcutaneous and in visceral adipose tissues of morbidly obese patients compared to non-obese subjects [27]. This might result in adipose tissue dysregulation since PPARβ/δ has anti-inflammatory effects in white adipose tissue. PPARβ/δ activation inhibits lipopolysaccharide (LPS)-induced cytokine expression and secretion by preventing nuclear factor (NF)-κB activation in adipocytes via the activation of mitogen-activated protein kinase (MAPK)–extracellular signal-regulated kinase (ERK)1/2 (MEK1/2) activation [28]. Furthermore, adipose tissue inflammation is characterized by increased infiltration and an altered polarization of the macrophages from the anti-inflammatory M2 phenotype towards the pro-inflammatory M1 phenotype, with PPARβ/δ a crucial signalling molecule that activates polarization towards the anti-inflammatory M2 phenotype [29]. Consistent with the effects of PPARβ/δ in adipose tissue, it has been reported that overweight patients with mixed dyslipidaemia who were administered the PPARβ/δ agonist MBX-8025 for 8 weeks presented favourable trends in their body fat percentage, lean body mass and waist circumference, although the differences did not reach statistical significance [30].

### 3.2. Dyslipidaemia

Atherogenic dyslipidaemia, often observed in patients with obesity, insulin resistance, metabolic syndrome and type 2 diabetes mellitus, is a significant risk factor for cardiovascular disease. This dyslipidaemia is characterized by the presence of low high-density lipoprotein (HDL) cholesterol levels, elevated triglyceride (TG)-rich very low-density lipoprotein (VLDL) amounts and an increased proportion of small and dense low-density lipoprotein (LDL) particles. It is presently accepted that atherogenic dyslipidaemia is initiated by insulin resistance through the overproduction of TG-rich VLDL [31]. Under conditions of insulin resistance, adipose tissue lipolysis is enhanced, leading to an increase in plasma non-esterified FA (NEFA). The subsequent increase in the flux of NEFA into the liver overcomes the oxidative capacity of hepatocytes and NEFA are then esterified for TG production, causing hepatic steatosis and VLDL over secretion in the plasma [31]. Finally, in the presence of hypertriglyceridemia, the cholesterol-ester content of LDL and HDL decreases, whereas these lipoproteins are enriched in their TG content through the activity of cholesteryl ester transfer protein. These TG-enriched particles are then hydrolysed by hepatic lipase, leading to the formation of small, dense LDL and to the decrease in HDL-cholesterol levels [31].

**Figure 2:**
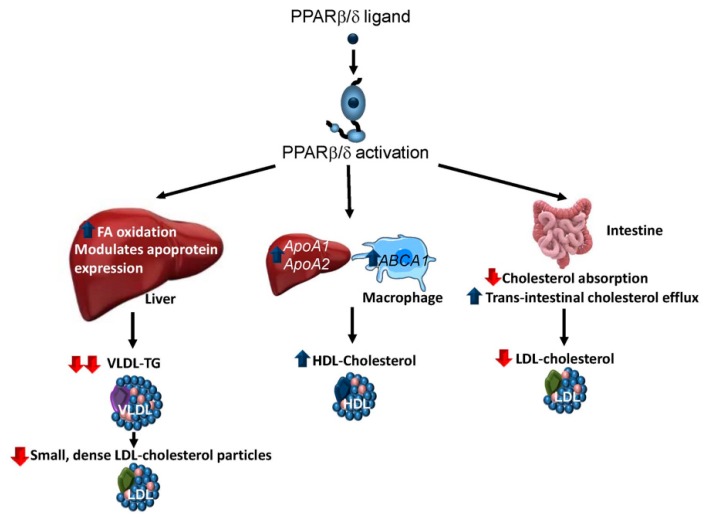
Effects of PPARβ/δ activation in dyslipidaemia. PPARβ/δ activation ameliorates atherogenic dyslipidaemia by reducing the amounts of very low-density lipoprotein (VLDL)-triglyceride (TG) and small dense low-density lipoprotein (LDL) particles and increasing the levels of high-density lipoprotein (HDL)-cholesterol. PPARβ/δ ligands reduce VLDL-TG by increasing hepatic fatty acid (FA) oxidation, which decreases the availability of this lipid for TG synthesis and changing the expression of several apoproteins. PPARβ/δ ligands increase HDL-cholesterol levels by elevating the amounts of the main apopoproteins of these lipoproteins (ApoA1 and ApoA2) in the liver and raising the levels of ATP-binding cassette A1 (ABCA1) in macrophages. Reduced LDL-cholesterol levels results from a decrease in cholesterol absorption and an increase in faecal excretion that are mediated by PPARβ/δ activation. Blue arrow: increases. Red arrow: decreases.

PPARβ/δ agonists show a strong TG-lowering action in vivo. Given that the main factor affecting hepatic TG secretion is FA availability, the hypotriglyceridaemic effect of PPARβ/δ activators has been attributed, at least in part, to their ability to induce FA β-oxidation in the liver [32] and other tissues [21,33] (Figure 2). In the liver, this role of PPARβ/δ involves the increased expression of the genes involved in FA oxidation via amplification of the lipin 1/PPARγ-coactivator 1α (PGC-1α)/PPARα signalling system and increased levels of the hepatic endogenous ligand for PPARα, 16:0/18:1-phosphatidylcholine [32]. Moreover, the increased FA β-oxidation caused by PPARβ/δ activators might be due to the activation of AMP kinase (AMPK), probably through an increase in the AMP:ATP ratio in hepatocytes [32]. Likewise, the effects of PPARβ/δ on the expression of several genes (*VldlR*, *ApoA5*, *ApoA4* and *ApoC1*) involved in lipoprotein metabolism can contribute to its hypotriglyceridaemic effect [32,34]. In accordance with these effects of PPARβ/δ, mice deficient in this receptor fed a high-fat diet (HFD) show increased plasma TG levels due to hepatic VLDL overproduction. Moreover, these mice also exhibited reduced activity of the enzyme lipoprotein lipase, which catalyses the hydrolysis of the TG component of circulating chylomicrons and VLDL.

PPARβ/δ agonists also increase plasma HDL-cholesterol levels and reduce LDL-cholesterol and NEFA levels in both animal models and humans [30,33,34,35,36]. The increase in plasma HDL-cholesterol levels following PPARβ/δ activation has been linked to an increased expression of the two major apolipoproteins of HDL, *ApoA1* and *ApoA2* [36], in the liver. In addition, PPARβ/δ activation increases the expression in macrophages of the reverse cholesterol transporter, ATP-binding cassette A1 (*ABCA1*) [36,37], which is crucial for the formation of HDL particles through its transport of cholesterol and phospholipid to apolipoprotein acceptors in the bloodstream. Furthermore, PPARβ/δ agonists also regulate the expression of hepatic phospholipid transfer protein (*Pltp*), which regulates the size and the composition of HDL and plays an important role in controlling plasma HDL levels [38]. More recently, it has been reported that the absence of intestinal PPARβ/δ abolishes the ability of its agonists to increase HDL-cholesterol plasma levels [22].

Regarding the reduction in LDL-cholesterol levels, PPARβ/δ agonists have been shown to decrease the efficiency of intestinal cholesterol absorption possibly by reducing the intestinal abundance of the cholesterol absorption protein, Niemann-Pick C1-like 1 (NPC1L1) [39]. Furthermore, PPARβ/δ activation also stimulates faecal excretion of cholesterol in mice, primarily by the two-fold increase in trans-intestinal cholesterol efflux [40], a non-hepatobiliary-related route that transports cholesterol from the blood to the intestinal lumen directly via enterocytes.

The assessment of PPARβ/δ agonists in several small-scale clinical trials mainly for the treatment of atherogenic dyslipidaemia has confirmed that these drugs reduce plasma TG levels, increase the amounts of HDL-cholesterol and decrease the levels of small dense LDL particles in humans, indicating that treatment with these drugs initiates a transition towards a less atherogenic lipoprotein profile [30,37,41,42,43].

### 3.3. Type 2 Diabetes Mellitus

More than 90% of patients with type 2 diabetes mellitus are overweight or obese, since obesity is associated with insulin resistance. Insulin resistance, which is defined as a defect in the ability of insulin to drive glucose into its target tissues, predicts and precedes the development of type 2 diabetes mellitus [44]. However, patients with insulin resistance do not develop hyperglycaemia and type 2 diabetes mellitus until the pancreatic β cells fail to secrete sufficient amounts of insulin to meet the increased metabolic demand for this hormone. Adipose tissue expansion in obese individuals releases increased amounts of NEFAs, hormones, pro-inflammatory cytokines and other factors that contribute to the development of insulin resistance. Most of these molecules cause a chronic low-level inflammation, which contributes to insulin resistance and type 2 diabetes mellitus [45].

**Figure 3:**
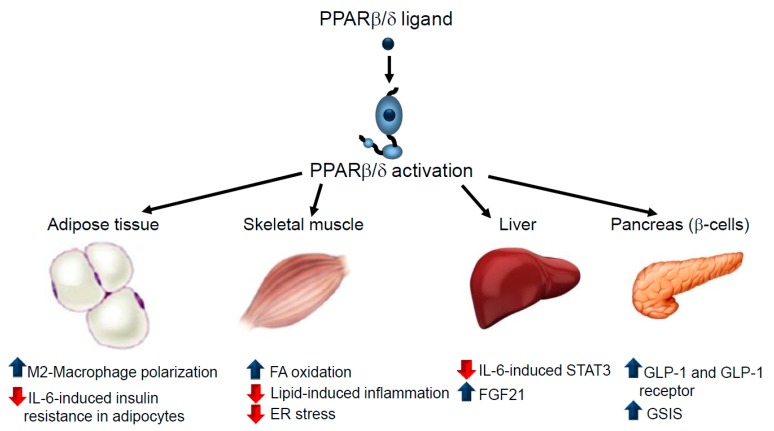
Effects of PPARβ/δ in type 2 diabetes mellitus. This figure depicts the effects of PPARβ/δ ligands in adipose tissue, skeletal muscle, the liver and pancreatic β cells that contribute to the attenuation of type 2 diabetes mellitus. In adipose tissue, PPARβ/δ activation switches macrophage polarization towards the anti-inflammatory M2 phenotype and prevents IL-6-induced insulin resistance by inhibiting STAT3. In skeletal muscle, PPARβ/δ ligands induce FA oxidation, reducing their availability for the synthesis of deleterious complex lipids involved in inflammation and prevent endoplasmic reticulum (ER) stress by activating AMPK. PPARβ/δ activation in hepatocytes blocks the effects of IL-6 by inhibiting the STAT3 pathway through several mechanisms and increasing FGF21 levels. PPARβ/δ activators promote the beneficial effects of GLP-1 in the pancreas and enhance GSIS.ER, endoplasmic reticulum; FA, fatty acid; GLP-1, glucagon-like peptide 1; GSIS, glucose-stimulated insulin secretion; IL-6, interleukin 6; STAT3, signal transducer and activator of transcription 3. Blue arrow: increases. Red arrow: decreases.

PPARβ/δ agonists improve glucose tolerance and insulin sensitivity in animal models [20,46]. The antidiabetic effects of these drugs are exerted in different tissues. For instance, macrophage infiltration into adipose tissue and polarization towards the pro-inflammatory M1 phenotype promotes inflammation and correlates with the degree of insulin resistance [47]. As mentioned above, PPARβ/δ activates polarization towards the anti-inflammatory M2 phenotype in macrophages [29] (Figure 3). In accordance with this, myeloid-specific PPARβ/δ^−/−^ mice show adipocyte dysfunction and insulin resistance [29]. Interestingly, a link exists between metabolism and function in macrophages. Thus, M2 macrophages require oxidative metabolism for their responses, whereas M1 macrophages depend on aerobic glycolysis [48,49]. In fact, blocking oxidative metabolism leads to the polarization of macrophages from the M2 to the M1 phenotype. Similarly, forcing oxidative metabolism in an M1 macrophage potentiates the M2 phenotype [50,51]. Given that PPARβ/δ activation increases β-oxidation in macrophages [52], this effect might also contribute to the polarization to the M2 phenotype caused by the agonists of this receptor. Macrophages also play a key function in a specialized phagocytic process called efferocytosys [53] that contributes to promoting the resolution of inflammation and PPARβ/δ activation enhances this process [53,54]. Since a defective efferocytosys has emerged as a causal factor in the etiopathogenesis of atherosclerosis [55], the increase in this process caused by PPARβ/δ activation might contribute to its beneficial effects in atherosclerosis.

Interleukin (IL)-6 is one of the inflammatory mediators released by adipose tissue that correlates most strongly with obesity and insulin resistance, predicting the development of type 2 diabetes mellitus [56]. PPARβ/δ activation prevents IL-6-induced insulin resistance by inhibiting the signal transducer and activator of transcription 3 (STAT3) pathway in adipocytes, whereas this pathway is over activated in PPARβ/δ-null mice compared to wild-type animals [57].

Skeletal muscle is the primary site of insulin resistance in obesity and type 2 diabetes mellitus since it displays the highest level of insulin-stimulated glucose utilization [5]. Increased plasma levels of saturated NEFAs, caused by the expansion of adipose tissue, promote inflammation and insulin resistance through several mechanisms: the synthesis of FA-derived complex lipids such as diacylglycerol and ceramides; the impairment of the function of cellular organelles (endoplasmic reticulum [ER] stress and mitochondrial dysfunction); and the activation of pro-inflammatory pathways through membrane receptors, such as toll-like receptor 4 (TLR4). PPARβ/δ activation the decrease in insulin sensitivity by suppressing the FA-induced increase in diacylglycerol levels and the subsequent activation of protein kinase C (PKC)θ and NF-κB by enhancing the expression of the genes involved in FA oxidation via PGC-1α and by increasing AMPK phosphorylation [58,59]. Furthermore, PPARβ/δ overexpression in the skeletal muscle of mice has been reported to promote the interaction between PPARβ/δ and AMPK, which enhances glucose uptake, FA oxidation and insulin sensitivity [60]. PPARβ/δ agonists also prevent palmitate-induced ER stress in myotubes through a mechanism involving AMPK activation [61]. Overall, it is believed that PPARβ/δ activation in skeletal muscle produces changes that resemble the effects of exercise training [61], making it a potential candidate for mimicking the effects of exercise to treat metabolic diseases [62].

In the liver, IL-6 induces insulin resistance by activating STAT3-suppressor of cytokine signalling 3 (SOCS3) pathway [63]. We have previously reported that PPARβ/δ activation prevents the attenuation of the insulin signalling pathway in human liver cells by preventing IL-6-induced STAT3 activation through a mechanism that inhibits ERK1/2 phosphorylation and suppresses the reduction in phospho-AMPK levels [64]. More recently, the inhibitory effect of PPARβ/δ on STAT3 was confirmed, with a new mechanism described involving a T cell protein tyrosine phosphatase 45 (TCPTP45) isoform [65]. According to this recent study, short-term PPARβ/δ activation prevents IL-6-induced insulin resistance as a result of PPARβ/δ forming a complex with nuclear TCPTP45 and retaining it in the nucleus, thereby deactivating the STAT3-SOCS3 signalling [65]. Fibroblast growth factor 21 (FGF21) is a liver-derived circulating hormone that has emerged as an important regulator of glucose and lipid metabolism, making it a promising agent for the treatment of insulin resistance and type 2 diabetes mellitus [66]. Since PPARβ/δ activators increase the plasma levels of FGF21 in humans [67], some of the antidiabetic effects of these drugs might be mediated by the increased levels of this protein.

β-cell failure, a result of the progressive decline in pancreatic β cell function and mass, impairs insulin secretion and contributes to the development of type 2 diabetes mellitus [68]. PPARβ/δ activation in the small intestine potentiates the production of glucagon-like peptide (GLP)-1, which preserves β cell morphology and function, thereby increasing systemic insulin sensitivity [69]. This is consistent with a recent study reporting that intestinal PPARβ/δ protects against diet-induced obesity and insulin resistance [26]. Moreover, PPARβ/δ activation protects pancreatic β cells from palmitate-induced apoptosis by upregulating the expression of the receptor for GLP-1 [70]. PPARβ/δ agonists also increase mitochondrial oxidation in β cells, enhance glucose-stimulated insulin secretion (GSIS) from pancreatic islets and protect GSIS from the adverse effects of prolonged FA exposure [71]. In fact, PPARβ/δ is critical for the expression of the genes involved in mitochondrial function and consequently ATP production, in β cells, which is required for GSIS [72].

### 3.4. Non-Alcoholic Fatty Liver Disease (NAFLD)

NAFLD encompasses a spectrum of liver disorders ranging from simple steatosis (non-alcoholic fatty liver, NAFL) to non-alcoholic steatohepatitis (NASH) and liver fibrosis. It is closely linked to obesity and metabolic syndrome, predisposing susceptible individuals to cirrhosis, hepatocellular carcinoma and cardiovascular disease [73]. At present, there are no approved pharmacological therapies for NAFLD. In animal models, long-term treatment with PPARβ/δ agonists attenuates hepatic steatosis by enhancing FA oxidation, reducing lipogenesis and improving insulin sensitivity [74,75,76,77]. PPARβ/δ activation and overexpression inhibit lipogenesis in hepatocytes by inducing the expression of insulin-induced gene-1 (*INSIG-1*), an ER protein that blocks the activation of sterol regulatory element-binding protein-1 (SREBP-1), a pivotal transcription factor controlling lipogenesis in hepatocytes [77]. However, short treatments with PPARβ/δ agonists might result in a transient increase in hepatic TG levels [78] but without hepatotoxicity, since PPARβ/δ increases the number of monounsaturated FAs but reduces the levels of saturated FAs [79]. In addition, PPARβ/δ might affect hepatic TG levels by regulating the abundance of the VLDL receptor [80]. In humans, PPARβ/δ agonists reduce hepatic fat content and elicit improvements in the plasma markers of liver function [30,33]. These and additional findings point to PPARβ/δ, similarly to PPARα, as a master regulator of hepatic intermediary metabolism. During fasting conditions, hepatic metabolism is programmed to oxidize FA and both, PPARα and PPARβ/δ are thought to promote ketogenesis by inducing FGF21 [67] and the expression of genes involved in fatty acid oxidation [81] in rodents. Interestingly, as mentioned before, PPARβ/δ activation in mice increases the hepatic levels of the hepatic endogenous ligand for PPARα, 16:0/18:1-phosphatidylcholine, leading to amplification of the PGC-1α-PPARα pathway [32], suggesting the presence of a cooperation between both nuclear receptors in the regulation of hepatic metabolism.

The progression from NAFL to NASH involves the development of inflammation and signs of hepatocellular damage [65]. Both the hepatic expression of inflammatory genes [74,76,82] and hepatic ER stress [76], which contribute to the activation of inflammatory pathways, are reduced by PPARβ/δ ligands. This hepatoprotective effect of PPARβ/δ ligands might also involve Kupffer cells, resident liver macrophages that play a critical role in maintaining liver functions. Hematopoietic deficiency of PPARβ/δ selectively impairs the alternative activation of Kupffer cells in obese mice, leading to reduced oxidative metabolism and hepatic dysfunction [83]. Additional studies are required to conclusively determine the effects of PPARβ/δ agonists on liver fibrosis due to the inconsistent results currently reported in the literature [17,84].

## 4. Safety of PPARβ/δ Agonists

Despite the promising data of PPARβ/δ agonists in metabolic disorders in preclinical studies, the discovery that mice treated with GW501516 developed adenocarcinoma [85] halted further development of this drug and undermined the potential use of these drugs in human therapeutics. However, subsequent attempts to assess the role of PPARβ/δ in cancer have demonstrated that this receptor both inhibits and promotes tumorigenesis, as it has been extensively reviewed previously [10,86,87], becoming one of the most controversial effects of PPARβ/δ. The conflicting results about PPARβ/δ in cancer might indicate that the activity of this receptor in cancer development is influenced by the mutational status of the tumour cell and the tumour environment [10]. Moreover, it has been proposed that the high-level expression of PPARβ/δ in normal cells suggest an antitumour effect for this receptor but the reduction in its expression or the presence of endogenous antagonists or inverse agonists might lead to a protumorigenic role for PPARβ/δ [86].

Although there are only a few clinical trials assessing the safety of PPARβ/δ agonists, the administration of these drugs to humans seems to be safe and generally well-tolerated, at least for the short periods evaluated [30]. Thus, no study subjects were withdrawn because of adverse effects from a study evaluating the administration of seladelpar for 8 weeks to dyslipidaemic overweight patients at doses showing beneficial metabolic effects [30]. Treatment with this PPARβ/δ agonist slightly but significantly decreased red blood cell count, haemoglobin and haematocrit. In a more recent study, the effect of seladelpar was assessed in patients with primary biliary cholangitis [88]. Drug treatment for 12 wk. elicited anti-cholestatic effect but three patients showed rapidly reversible alanine aminotransferase elevations and the study was interrupted before completion. The authors of the study suggest that this effect might be specific for these patients with primary biliary cholangitis due to the biliary excretion of seladelpar ant its metabolites, leading to increased hepatic drug concentrations.

## 5. Conclusions

Over the past 20 years, a substantial body of preclinical evidence has demonstrated that PPARβ/δ activation is a promising therapeutic strategy for treating obesity-associated co-morbidities. This has led to the assessment of PPARβ/δ agonists in clinical trials, although these studies on the efficacy and safety of PPARβ/δ agonists in humans are scarce. Safety issues have been raised regarding the role of PPARβ/δ ligands in carcinogenesis [87]. However, there are conflicting findings on the role of PPARβ/δ as a tumour suppressor or tumour promoter [87], the latter being mostly observed in animal models. Further studies are required to obtain conclusive data on the role of PPARβ/δ in human cancer given that although mouse models are invaluable tools for investigating basic tumour biology, they show significant limitations when compared to human beings. For instance, PPARs are expressed at lower levels in human than in rodent cells and gene expression is also regulated differently by PPARs in human and rodent cells [9]. These differences might explain why long-term treatment with PPARα ligands have been shown to induce carcinogenesis in rodents but carcinogenesis has not been observed in humans treated with the PPARα agonists fibrates for dyslipidaemia over the decades [89]. However, this needs to be studied for PPARβ/δ too.

In summary, although further studies are required to confirm the safety of PPARβ/δ ligands, these drugs have demonstrated that modulation of PPARβ/δ activity shows efficacy in preclinical studies and in a few clinical trials in the treatment of dyslipidaemia, type 2 diabetes mellitus and NAFLD.
